# The novel epiligament theory: differences in healing failure between the medial collateral and anterior cruciate ligaments

**DOI:** 10.1186/s40634-021-00440-0

**Published:** 2022-01-14

**Authors:** Georgi P. Georgiev, Manasi Telang, Boycho Landzhov, Łukasz Olewnik, Svetoslav A. Slavchev, Robert F. LaPrade, Kacper Ruzik, R. Shane Tubbs

**Affiliations:** 1 Department of Orthopedics and Traumatology, University Hospital Queen Giovanna-ISUL, Medical University of Sofia, Sofia, Bulgaria; 2 Department of Anatomy, Histology and Embryology, Medical University of Sofia, Sofia, Bulgaria; 3 Department of Anatomical Dissection and Donation, Chair of Anatomy and Histology, Medical University of Lodz, Łódź, Poland; 4 University Hospital of Orthopedics “Prof. B. Boychev”, Medical University of Sofia, Sofia, Bulgaria; 5 Twin Cities Orthopedics, Edina, Minnesota USA; 6 Department of Normal and Clinical Anatomy, Interfaculty Chair of Anatomy and Histology, Medical University of Lodz, Lodz, Poland; 7 Department of Anatomical Sciences, St. George’s University, True Blue, Grenada; 8 Department of Neurosurgery, Tulane University School of Medicine, New Orleans, Louisiana USA; 9 Department of Neurology, Tulane University School of Medicine, New Orleans, Louisiana USA; 10 Department of Structural and Cellular Biology, Tulane University School of Medicine, New Orleans, Louisiana USA; 11 Department of Surgery, Tulane University School of Medicine, New Orleans, USA

**Keywords:** Epiligament, Medial collateral ligament, Anterior cruciate ligament, Healing, Novel theory

## Abstract

According to current literature, 90% of knee ligament injuries involve the medial collateral ligament or the anterior cruciate ligament. In contrast to the medial collateral ligament, which regenerates relatively well, the anterior cruciate ligament demonstrates compromised healing. In the past, there were numerous studies in animal models that examined the healing process of these ligaments, and different explanations were established. Although the healing of these ligaments has been largely investigated and different theories exist, unanswered questions persist.

Therefore, the aim of this article is 1) to review the different historical aspects of healing of the medial collateral ligament and present the theories for healing failure of the anterior cruciate ligament; 2) to examine the novel epiligament theory explaining the medial collateral ligament healing process and failure of anterior cruciate ligament healing; and 3) to discuss why the enveloping tissue microstructure of the aforementioned ligaments needs to be examined in future studies.

We believe that knowledge of the novel epiligament theory will lead to a better understanding of the normal healing process for implementing optimal treatments, as well as a more holistic explanation for anterior cruciate ligament healing failure.

## Introduction

One of the most commonly injured ligaments in the knee is the medial collateral ligament (MCL) [1–4]. Most MCL injuries are the consequence of external rotation, valgus loading, or a combined force vector in sporting activities such as football, skiing, and ice hockey. They are mostly isolated and occur predominantly in young athletes [3, 5]. They are typically associated with mediolateral instability, especially during cutting or pivoting maneuvers [5]. It is now well established that most knee ligament injuries involve the MCL or the anterior cruciate ligament (ACL). The incidence of MCL injury has increased over recent decades and is frequently encountered in modern sports medicine [5]. After injury, the ligaments do not heal by regeneration but by formation of scar tissue, similar to other wound healing models [6–8]. Many studies have shown that while the MCL can heal fairly well, it cannot be fully restored. Therefore, different treatment options, such as tissue engineering approaches, nonsteroidal anti-inflammatory drugs, local corticosteroids, hyperbaric oxygenation, growth factors, ultrasound or electrical stimulation, laser therapy, and gene therapy have been attempted [9–12]. Unlike the MCL, the ability of the ACL to heal spontaneously is inadequate. In the past, numerous experiments in different animal models have been performed, and different theories have been proposed to explain the healing differences in these ligaments; however, questions regarding the superior healing process of the medial collateral ligament and its failure in injuries of the anterior cruciate ligament still exist [1, 5, 13–16]. It should be noted that all the morphological explanations about the healing process of these ligaments in animal models investigate only the extracellular matrix in both normal and injured ligaments, and none of them considers the enveloping tissue of the ligament, termed epiligament (EL) [3, 11, 12, 17].

After extensive research on epiligament morphology of the MCL in rats and humans in both normal conditions and injury, as well as investigation of the same tissue in the ACL and comparison between these two commonly injured ligaments, a novel EL theory was proposed by Georgiev et al. [6–8, 11, 12, 18–22].

The aim of the current paper is to review the current literature concerning the knowledge of commonly injured ligaments of the knee and the differences between MCL and ACL healing. We discuss in detail how this new theory clearly explains the worsened capacity for healing of the ACL. We also emphasize why future investigations for better advances in ligament repair treatment should be directed to the epiligament.

### MCL anatomy

The MCL is an intricate structure that acts as the primary static stabilizer of the knee joint, counteracting rotation caused by a valgus force [1, 23]. It comprises three constituents: the superficial MCL (sMCL), the deep MCL (dMCL) and the posterior oblique ligament (POL) [5, 24]. The largest ligament in the medial aspect of the knee is the sMCL, which connects the medial femur and tibia through one femoral and two tibial attachments [5]. The thickened medial portion of the joint capsule is represented by the dMCL and has two parts, the meniscofemoral and meniscotibial ligaments [5]. The posteromedial aspect of the joint capsule is attached to and reinforced by fibrous extensions from the main common tendon of the semimembranosus, which constitute the POL [5, 24].

### ACL anatomy

The ACL arises from the distal femur and attaches to the anterior intercondylar area of the tibia [25]. It is composed of two bundles, anteromedial and posterolateral, with different tibial attachments [26–28]. Excessive anterior translation and internal rotation of the tibia relative to the femur is resisted by the ACL [14, 25]. Studies indicate that the anteromedial bundle is primarily responsible for resistance to anterior tibial translation, while the posteromedial bundle contributes to the control of tibial rotational laxity [26].

### Ligament microstructure

Ligaments are hypocellular and hypovascular structures built of dense regular connective tissue [29–31]. Collagen is the most widespread extracellular component of soft connective tissue and is the major tensile-bearing element [6, 7, 31, 32]. The endoligament is sheath of connective tissue that covers the collagen fibers in the ligament proper, which are organized into fascicles [6–8, 11, 12, 22, 33]. Collagen constitutes approximately 75% of the dry weight of ligaments, the predominant type being type I, which accounts for nearly 85% of the total collagen of ligaments and is chiefly responsible for their tensile strength [6, 7, 32, 34]. The remaining 15% includes types III, V, VI, XI, and XIV [34]. Type III is involved in ligament repair, and its synthesis is significantly increased after grade III ligament injuries [9, 30, 34]. The role of collagen type III in proper ligament recovery has been discussed previously [30, 35]. Its synthesis swiftly increases during the first stages of ligament healing and during ligamentization after tendon grafting, exceeding the level of synthesis of type I. Rates typically return to normal by 52 weeks after injury [30, 35]. Hauser et al. [34] found that after injury, fibroblasts mainly synthesize collagen type III. Considering the large number of fibroblasts in the EL, it is reasonable to suggest that they are chiefly responsible for the upregulation of collagen type III.

According to Yang et al. [36], collagen type III is also essential for generating tissue matrix, fetal tissue matrix, and scars. These authors proposed that the ability of collagen type III to crosslink by disulfide bridges contributes to its favorable deposition in sites of tissue regeneration. Amiel et al. [37] also established that collagen type III increased during ligamentization after tendon grafting.

On the other hand, collagen type V engages in the organization of collagen type I fibrils and the regulation of their diameters, which also occurs during ligament healing [6, 7, 35, 38]. According to Breuls et al. [38], type V collagen fibrils participate in the regulation of extracellular matrix modeling and remodeling by controlling collagen fibril initiation. Collagen type XIV is involved in linear fibril growth [30].

Collagen fibers in ligaments are organized into fascicles enveloped by a thin connective tissue sheath known as the endoligament. The endoligament in turn is connected to a more vascular connective tissue layer that covers the entire ligament, termed the epiligament (EL) [8, 20, 33].

### Structure of the epiligament

Surface layers of connective tissue are characteristically associated with bone, cartilage, striated muscle, nerves, and tendons. These layers are termed periosteum, perichondrium, epimysium, epineurium, and epitendon, respectively [39]. Bray et al. [40], in ‘Fine vascular anatomy of adult rabbit knee ligaments,’ provided the first definition of ‘epiligament’ (epi- [Greek – on or upon]; ligament [Latin – *ligare*, to bind]). This structure has been described as ‘surrounding adherent connective tissue removed simultaneously with the ligament but … grossly distinguishable from ligament tissue proper’ [40]. Later, Chowdhury et al. [33] also examined the external surface of the EL of the MCL in rabbits and described two types of cells, fibroblasts and spinous adipocytes. Apart from covering the ligament tissue, the EL merges with the periosteum at the sites of ligament insertion [29]. In contrast to the ligament, the EL contains multiple cell types such as fibroblasts, fibrocytes, adipocytes, and blood vessels [6–8, 11, 12, 18–20, 33, 41]. Georgiev et al. [21] presented the ultrastructural characteristics of these cells. Fibroblasts have been described as large and well formed; they display a very delicate chromatin structure with a prominent nucleolus. The cytoplasm contains free ribosomes, polysomes, a well-presented rough endoplasmic reticulum, a poorly developed Golgi apparatus, spherical mitochondria, and single lysosomes. The second cell type, spindle-shaped fibrocytes, were described as having large vacuoles and eccentric, flat nuclei surrounded by a basal lamina and rough endoplasmic reticulum. In the intercellular space, Georgiev et al. also found collagen fibers with multiple orientations, as well as myelinated and unmyelinated nerve fibers and blood vessels. Similar ultrastructural characteristics of fibroblasts and fibrocytes, as reported above, were confirmed by Georgiev et al. [8] in humans. They finally concluded due to the ultrastructural characteristics that fibroblasts might be involved in differentiation, phagocytosis, and collagen synthesis; the authors also hypothesized that single collagen fibers or those grouped in bundles may respond to ligament tension in different directions [8, 21]. With the aim of obtaining more detailed knowledge of the MCL and ACL ELs and comparing it in rats, Iliev et al. [32] found a statistically significant difference in the number of cells per mm^2^ in the EL of the two ligaments, with a greater number in the MCL. Furthermore, the EL contains an abundance of blood vessels and sensory/proprioceptive nerve elements, which form a complex network [6–8, 11, 12, 18–20, 33]. It has been proposed that the EL has a role in ligament growth and healing and may control water and metabolite influx into the ligament [33, 40]. Several studies have pointed toward the EL as a donor of fibroblasts and other connective tissue cells, progenitor cells, and blood vessels, which migrate toward the body of the ligament via the endoligament and are crucial for ligament repair [6–8, 11, 12, 18–21, 41]. These studies clearly indicate that during early healing, EL tissue formed new granulation tissue after injury via the endoligament and thus is the main donor of cells and blood vessels for the repair process. Georgiev et al. [12] statistically analyzed the number of cells in the EL-ligament scar, which demonstrated no difference between spontaneous healing and healing after suture application. The reported histological data on the EL’s main role in ligament repair and the proposal of a new EL theory could be used as a basis for the development of new treatment regimens with improved patient outcomes.

The collagen fibrils in the EL mainly comprise collagen types III and V [6, 7, 9, 22, 32]. Collagen type III is integral to ligament repair [9, 30, 34]. Collagen type V is also associated with ligament recovery, and the intensity of its expression corresponds to the diameter of collagen fibrils [35]. In the MCL and ACL, the immunohistochemical expression of the aforementioned collagen types was stronger in the EL than in the ligament proper and was greater in the EL of the MCL than in the ACL [32]. Fibroblasts are also responsible for the synthesis of matrix metalloproteinases (MMPs), decorin, fibronectin, and fibromodulin, which are involved not only in the degradation of the ligament after injury but also in subsequent cell proliferation and ligament remodeling [6–9, 11, 12, 18–22]. Georgiev et al. [20] reported that the enzymatic activity of MMP-2 and MMP-9 was greater in the EL of the MCL than in the same structure of the ACL and speculated the important role of these enzymes in the normal function and difference in the healing potential of these ligaments.

### Ligament healing morphology

In a dog model, O’Donoghue et al. [42] reported that surgical treatment led to a decrease in the amount of newly synthesized tissue and accelerated collagenisation in the healing process, thus improving morphological characteristics. Hart and Dahners [43] and Hildebrand and Frank [44] found no statistically significant difference in improved endurance of the MCL between nonoperative treatment and treatment by suturing. According to Chimich et al. [45], bringing the ends of the ruptured ligament closer together has a definite advantage during the granulation and remodeling stages since it improves the macroscopic, histological, and biomechanical properties of the ligament. According to the authors, the shorter distance between the two ends approximated by suturing is associated with faster filling of the space between them with newly synthesized tissue and with more rapid remodeling. Nevertheless, in an experimental rabbit model, Chimich et al. [45] demonstrated that there was no statistically significant difference in the strength of the ligament after 40 weeks between animals treated surgically and those treated conservatively. These authors also noted a number of similarities between the models of the two types of treatment: healing is mediated by granulation tissue, which is macroscopically and histologically different from normal tissue; approximately 3 weeks after injury, the neoligament is characterized by hypercellularity and is mixed with other healthy tissues; in comparison with the contralateral ligament at 14 weeks, the laxity of the injured ligament appears restored; the newly formed connective tissue is shortened, which is also observed in other connective tissue models; remodeling of the tissue continues over time, but the macro- and microscopic appearances of a regenerating tissue are evident even after 40 weeks; in both models, approximately 65% of the strength of the ligament is restored after the 40th week compared to the contralateral ligament.

Loitz-Ramage et al. [46] compared the outcomes of conservative and operative treatments in a rabbit model over a longer period of time. The authors compared the results of treating an 8 mm gap between the ends of the torn ligament with or without a Z-suture and found that reducing the distance to 4 mm using the suture generated greater strength than was achieved at 8 mm. Extrapolating this result to clinical practice, the authors stated that patients with longer distances between the two ends of the torn ligament, for instance in those patients in which adjacent bones are dislocated or ligament ends are retracted, could restore normal function through low or moderate loading, but there is a significant risk of re-rupture in the event of heavy loading. Using their rabbit model, McDougall et al. [47] reported that levels of angiogenesis during the sixth week after surgical treatment corresponded to those in control animals that had undergone placebo surgery. The authors suggested that operative treatment with suture application could have accelerated the healing process so much that angiogenesis had already been reversed; otherwise, the repositioning of the ends of the injured ligament might have hindered neovascularization.

Ishiguro et al. [48] reported the presence of promatrix metalloproteinase-9-positive cells in the perivascular area of the ruptured ACL and promatrix metalloproteinase-2-positive cells between irregular collagen bundles in the stumps of this ligament. The authors could not determine whether the positive reaction of these MMPs was due to rapid degradation or the result of prior degradative changes. Creighton et al. [10] suggested that surgical treatment would decrease the maximum distance between the two ends of the torn ligament with the intent of improving healing if rupture of the ligament was incomplete. The tear would thus be shortened, and the ligament would be realigned in a state close to its anatomical one [47].

After comparing the expression of different MMPs in the MCL and the ACL, Zhou et al. [49] concluded that numerous MMPs could be associated with differences in healing potential. They reported that fluorescent MMP-2 activity was higher in the injured ACL than in the MCL, which could be one reason for ACL healing failure. Majima et al. [17] compared cyclic creep between surgically repaired and nonrepaired ligaments and found no significant difference; regardless of whether the ligament was operated on, creep was 3–4 times that of a normal ligament 6 weeks after injury. The authors concluded that acute ligament repair did not alter the potential of the healing complex to creep relative to controls, even at 6 weeks after injury. According to Tang et al. [50], MMP-2 expression in fibroblasts of the injured ACL was 6.3 times higher than that of the injured MCL. MMP-9, in contrast, was upregulated in the injured MCL but to a much lesser degree than in the injured ACL. Zhang et al. [51] reported higher mRNA levels of MMP-1, MMP-2, MMP-14, MMP-17, MMP-23A, MMP-23B and TIMP-4 in MCL fibroblasts than in ACL fibroblasts. Furthermore, it was suggested that the differential expression of MMPs between the MCL and ACL could partly account for the differential healing potentials of the two ligaments. Georgiev et al. [19] observed the distribution and expression of MMP-2 in normal rat tissue and during healing after acute injury. They showed that fibroblasts in the EL of the MCL normally generated low levels of MMP-2. After grade III injury, high levels of MMP-2 were expressed during early ligament healing. Georgiev et al. [12] were the first to describe the ultrastructural changes in the EL of the MCL during the first month of ligament healing after injury. On the eighth day after injury, intensive angiogenesis was observed in the EL; the granulation tissue between the transected regions was hypercellular and represented mainly by EL fibroblasts and progenitor cells migrating through the endoligament. On the sixteenth day, diminished angiogenesis in the EL was observed, with less distinguishable granulation tissue and hypercellular tissue but a better organization pattern of EL-ligament scarring; the cells of the EL also migrated through the endoligament. On the thirtieth day after injury, the healing process advanced, and the EL tissue was similar to controls. In conjunction with the light microscopic study, the authors also presented in detail the ultrastructural characteristics of the fibroblasts in the EL, with well-formed large nuclei and clearly visible heterochromatin; the cytoplasm had well-developed rough endoplasmic reticulum, poorly developed Golgi apparatus, free ribosomes, polysomes, mitochondria, phagocytic vacuoles and lysosomes. All these characteristics define these cells as metabolically active structures during the healing process. Finally, light and electron microscopic observations showed no difference in the structure of the ligament during spontaneous healing and after suture application, as indicated by the numbers of cells in the EL ligament scar tissue. According to the authors, the limitations of this animal model were as follows: (1) all injuries were induced by scalpel transection, not an ideal simulation of common traumatic injuries; (2) only the EL of the mid-substance of the MCLTCL was studied; (3) the distance between the transected edges was no more than 1.5 mm; and (4) the statistical analysis used nonparametric tests, which generally have lower power. To attempt to mitigate this, the authors used two types of tests, the Kruskal–Wallis H test and Mood’s median test.

### Different explanations of ACL healing failure

Much is known about the different healing potentials of the ACL and MCL [1, 5, 14–16]. The ACL has poor healing ability, in contrast to MCL [1, 5, 14]. However, the reasons for the difference are not entirely clear, but multiple explanations have been suggested.

One explanation points to the fact that the ACL has an intra-articular location and is exposed to synovial fluid, which inhibits fibroblast function [52]. Another attributes it to the different ultrastructures of the connective tissue cells in the MCL and ACL [53]. More recent studies showed differences in fibroblasts and their proliferative potential between the ACL and MCL [54]. It has been suggested that higher levels of nitric oxide produced by ACL cells cause collagen inhibition and proteoglycan synthesis [55]. Others have noted a stronger blood flow and more pronounced angiogenesis in the MCL after injury, followed by accelerated healing [56]. MMP-2, − 9, and − 13 expression is also reported to differ [20, 57]. The failure of cells and blood vessels to adequately bridge the gap between the ruptured ends of the ACL could be one reason for the unsatisfactory healing of this ligament [58]. Vavken and Murray [59] suggested that plasmin circulating in synovial fluid slows healing by breaking down the fibrin clot. Moreover, the intra-articular environment reduces biological and mechanical support by adjacent tissues, meaning that cells and blood vessels are less likely to bridge the gap between the ligament’s ruptured ends properly [59].

The existence of these theories explaining ACL failure healing has shown that this process is “multifactorial” and that no one theory can explain the reasons for an inadequate healing process. A novel theory amplifying the others, describing one single unit ligament complex (target for future treatment strategies) and that could be responsible for healing or healing failure, would be ideal.

### Novel epiligament theory

Several reports by Georgiev et al. [6–8, 11, 12, 18–22] have presented the EL as a donor of fibroblasts, progenitor cells, and blood vessels, which migrate via the endoligament toward the ligament body. The authors accept its key role in ligament function and healing. Their recent studies [12, 18, 19] of MCL injury models have revealed that the fibroblasts in the EL are not static but are responsible for synthesizing various types of collagen, matrix metalloproteinases, decorin, fibronectin, and fibromodulin. All these molecules are implicated in the degradation, proliferation, and remodeling of the ligament after trauma [12, 18, 19, 32].

After investigations of the EL in rats, this tissue has been studied in humans. To confirm its supposed role and to account for ACL healing failure, Georgiev et al. [22] presented the morphologies of the ELs of human MCL and ACL. The authors established that the EL of the MCL and ACL in humans was quite different from the morphology of the ligament substance, which confirmed data of EL morphology in the rat. The EL of the MCL and ACL was comprised of fibroblasts and fibrocytes, adipocytes, collagen fibers and neurovascular bundles. This morphological description confirms the similarity of the EL between humans and rats. Additionally, the number of cells enumerated during light microscopy revealed that fibroblasts in the EL of the MCL were greater than those in the EL of the ACL. After comparative quantitative analysis of the number of cells, the authors established that the mean number of cells in the EL of the MCL was 32% greater than that in the EL of the ACL; the EL passed through the endoligament to the ligament proper, and in comparing these structures, the authors established that the endoligament of the MCL contained 36% more cells than that of the ACL. A greater incidence of cells per mm^2^ in the EL was reported in the MCL than in the ACL. These data confirmed previous literature data describing the similar predominance of cells in the EL of the MCL as compared to the same structure of the ACL in the rat. After the presented quantitative analysis, Georgiev et al. [22] suggested that further reasons for the discrepancy in healing potential could be the differences in the expression of collagen types I, III, and V in the ELs and ligament proper.

After evaluation of the immunohistochemical expression of the aforementioned collages, the authors observed that in the EL, immunostaining for collagen type I is localized predominantly in the tunica media of the blood vessels and in the ligament proper of both ligaments, where immunoreactivity was expressed ubiquitously and appeared moderate. For procollagen type III, a positive immunoreactivity was observed in the adventitia of blood vessels and the periphery of adipocytes and was stronger in the EL of the MCL. Expression was also strong in the ligament proper of the MCL in contrast to the ACL, where it was low or absent. The authors report that collagen type V was detected in the tunica media of blood vessels and in the superficial layer of the EL in both ligaments. For precise evaluation of expression, a semiquantitative IHC profiler was performed. Finally, the authors reported that (1) there are fewer connective tissue cells in the EL of the ACL than in the MCL, and the difference is statistically significant; (2) the expression of collagen types I and V and procollagen type III is higher in the EL of the MCL than in the ACL; and (3) procollagen type III is also expressed in the ligament tissue of the MCL but not in that of the ACL under physiological conditions.

According to Georgiev et al. [22], fewer cells in the EL of the ACL than in the MCL in the healthy knee cannot ensure an adequate healing capacity. Moreover, the expression of collagen type I (accounting for ligament tensile strength), procollagen type III (integral to proper ligament healing), and collagen type V (organizing collagen type I fibrils and regulating their diameters) in fibroblasts is also lower in the EL of the ACL than in the MCL. All aforementioned variations in EL morphology and differences in fibroblast activity in the healthy knee, especially the expression of procollagen type III, provide additional explanations for the failure of ACL healing after trauma.

Finally, the authors concluded that the ELs of the MCL and the ACL are quite different from the ligaments proper. They contain abundant fibroblasts, fibrocytes, and adipocytes as well as neurovascular bundles. EL fibroblasts are not static cells and produce various quantities of collagen types I, III, and V. Immunologically, expression of the collagen types studied was higher in the EL than in the ligament proper and higher in the EL of the MCL than in the ACL. In addition, there was a statistically significant difference between the numbers of cells per square millimeter in the ELs of the two ligaments that was higher in the MCL. Thus, Georgiev et al. have presented new data on the structural and functional significance of EL tissue that suggests it could be responsible for the better healing capacity of the MCL over the ACL.

## Conclusion

In the current literature review, we present what is known about ligament healing in the MCL. We also present commentary on this topic to draw attention to the novel EL theory concerning a novel presentation of the healing process regarding the newly formed tissue filling the gap between the ends of the ruptured ligament. We emphasize that future investigations for better ligament restoration should be directed at the EL because it contains the major substrate of cells and blood vessels responsible for ligament healing. The novel theory, based on the number of cells and vessels and the distribution of collagens and matrix metalloproteinases, could simply and clearly explain the worsened capacity of ACL healing. This new theory further developed existing theories based on differences in fibroblasts, impaired blood flow and MMP expression. This could be a starting point for new treatment strategies. The future will reveal whether this novel hypothesis will be accepted by morphologists and knee surgeons and whether it will be embraced as another explanation of ACL healing failure.
